# Early Development and Orientation of the Acoustic Funnel Provides Insight into the Evolution of Sound Reception Pathways in Cetaceans

**DOI:** 10.1371/journal.pone.0118582

**Published:** 2015-03-11

**Authors:** Maya Yamato, Nicholas D. Pyenson

**Affiliations:** 1 Departments of Vertebrate Zoology and Paleobiology, National Museum of Natural History, Smithsonian Institution, Washington, District of Columbia, United States of America; 2 Department of Paleobiology, National Museum of Natural History, Smithsonian Institution, Washington, District of Columbia, United States of America; 3 Departments of Mammalogy and Paleontology, Burke Museum of Natural History and Culture, Seattle, Washington, United States of America; New York Institute of Technology College of Osteopathic Medicine, UNITED STATES

## Abstract

Whales receive underwater sounds through a fundamentally different mechanism than their close terrestrial relatives. Instead of hearing through the ear canal, cetaceans hear through specialized fatty tissues leading to an evolutionarily novel feature: an acoustic funnel located anterior to the tympanic aperture. We traced the ontogenetic development of this feature in 56 fetal specimens from 10 different families of toothed (odontocete) and baleen (mysticete) whales, using X-ray computed tomography. We also charted ear ossification patterns through ontogeny to understand the impact of heterochronic developmental processes. We determined that the acoustic funnel arises from a prominent V-shaped structure established early in ontogeny, formed by the malleus and the goniale. In odontocetes, this V-formation develops into a cone-shaped funnel facing anteriorly, directly into intramandibular acoustic fats, which is likely functionally linked to the anterior orientation of sound reception in echolocation. In contrast, the acoustic funnel in balaenopterids rotates laterally, later in fetal development, consistent with a lateral sound reception pathway. Balaenids and several fossil mysticetes retain a somewhat anteriorly oriented acoustic funnel in the mature condition, indicating that a lateral sound reception pathway in balaenopterids may be a recent evolutionary innovation linked to specialized feeding modes, such as lunge-feeding.

## Introduction

The auditory system of cetaceans diverges from its nearest mammalian relatives in two profound ways. First, originating from terrestrial ancestors, the auditory hardware of cetaceans has been completely modified for an obligate aquatic existence [1]. Cetaceans do not have external pinnae, their ear canals are vestigial, and the bones housing the middle and inner ears are separated from the skull as a dense tympanoperiotic complex, which reduces bone conduction under water [2,3]. Second, the two living clades of cetaceans evolved to opposite extremes in auditory specializations: odontocetes (toothed whales) are high-frequency specialists capable of ultrasonic echolocation [4,5] while filter-feeding mysticetes (baleen whales) are low-frequency specialists that use infrasonic sounds for long distance communication [6,7].

In odontocetes, the outer ear functionally consists of acoustic fats, or unique lipids found within the hollowed portions of the mandible [8]. These fats extend posteriorly and attach to the tympanoperiotic complex over a broad area, including a thin tympanic plate [9,10] and a cone-shaped feature on the lateral face of the tympanoperiotic complex named the “*schalltrichter* [sound-funnel]” or the “ear trumpet” [2,11,12]. Although this acoustic funnel has not received much attention in the literature, finite element analyses of odontocete tympanoperiotics by Cranford et al. [12] suggest that this structure plays an important role in sound reception.

**Fig 1:**
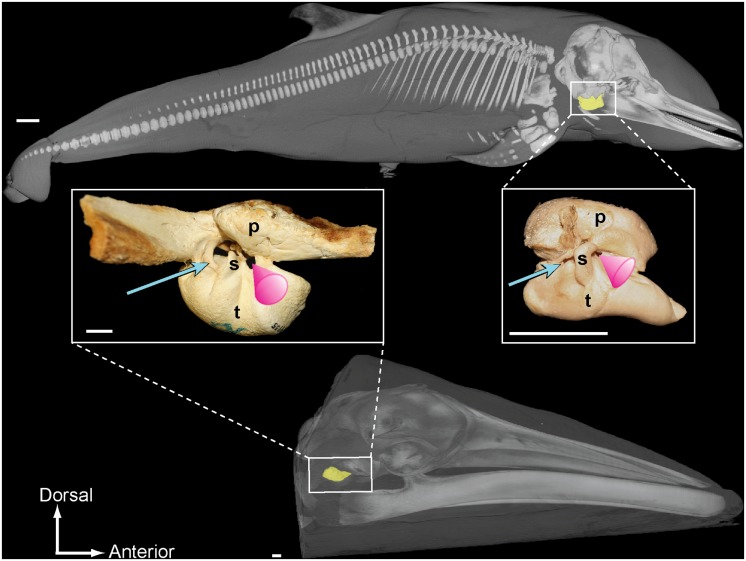
CT-based three-dimensional reconstructions of a toothed whale and baleen whale, right lateral view. Top: *Stenella attenuata* (USNM 504048). Bottom: *Balaenoptera acutorostrata*, juvenile specimen not part of this study. The tympanoperiotic complex houses the middle and inner ear structures, and is highlighted in yellow. The insert images are photographs of prepared tympanoperiotic complexes from mature individuals: *B*. *bonarensis* (USNM 504955) and *S*. *attenuata* (USNM 487880). The tympanic aperture is indicated by the blue arrow and the acoustic funnel is illustrated by the pink cone. Scale bar = 2 cm.

The acoustic funnel is separated from the tympanic aperture and the vestigial ear canal by the sigmoid process of the tympanic bone; therefore, the location of sound input for odontocetes is displaced anteriorly relative to the position of the tympanic aperture (Fig. 1). While sound reception mechanisms remain unclear in mysticetes, which do not have hollow mandibles filled with acoustic fats, there is new anatomical evidence suggesting that at least rorquals (Balaenopteridae) may also hear through fatty tissues associated with the ears [13]. These fatty tissues insert into the tympanoperiotic complex in an area that is homologous to the attachment site of odontocete acoustic fats, just anterior to the tympanic aperture and sigmoid process (Fig. 1; refer to [14–15] and S1 Text for anatomical terminology).

The morphological transformations involved in the early evolution of the cetacean ear, from terrestrial ancestors to obligate aquatic descendents, is well-documented in the fossil record (e.g., [16–20]). However, the evolution of underwater hearing in living clades, after the achievement of obligate aquatic life, remains poorly understood. Ontogenetic patterns in living cetaceans provide a crucial line of evidence for understanding the formation of the extreme ears of cetaceans. Most of the in-depth, classic investigations of fetal cetaceans were conducted before sound reception pathways were known (e.g., [21–23]), and the more recent studies are either limited in taxonomic coverage or focused on postnatal growth [24–34].

We investigated the developmental origin of the acoustic funnel in cetacean ears and charted their evolutionary implications by building an X-ray computed tomography (CT) dataset of various developmental stages (totaling 15 species of living cetaceans). These specimens mostly consisted of fluid-preserved, intact vouchers collected in association with commercial whaling operations in the early to mid-20^th^ century, along with samples from fisheries by-catch and strandings, which were all archived at the Smithsonian Institution. CT data provided crucial non-destructive insight into fetal stages that are difficult to observe and under-sampled relative to post-natal vouchers that dominate curated collections of cetacean material. Our findings show that the acoustic funnel develops early in the ontogeny of all cetaceans, and then diverges in anatomical orientation and morphology at late fetal stages of odontocetes and mysticetes. We also found that the orientation of the acoustic funnel correlates with previously described sound reception pathways for each clade. The phylogenetic distribution of these traits across living cetaceans (i.e., crown Cetacea) suggests that the acoustic funnel has been present at least since the Eocene-Oligocene transition (~34 million years ago), and that a laterally directed sound reception pathway in rorquals is an innovation likely linked to other cranial and mandibular specializations for lunge-feeding.

## Materials and Methods

We examined a total of 56 fetal cetacean specimens, including 3 families of mysticetes (7 species; 32 specimens) and 7 families of odontocetes (8 species; 24 specimens). All specimens were from the Smithsonian Institution’s National Museum of Natural History (NMNH; Washington, D.C., U.S.A.); USNM specimen numbers are listed in Table 1. These specimens included fluid-preserved, intact specimens (n = 45; see Table 1) and prepared, osteological specimens (n = 11; indicated by asterisks in Table 1). All fluid-preserved specimens and one osteological specimen (USNM 269156) were CT scanned at the NMNH using a Siemens Somatom Emotion 6 scanner at slice thicknesses ranging from 0.1 mm for the smaller specimens and 0.6 mm for the largest specimens. The computer software ORS Visual SI (Object Research Systems, Inc., Montréal, Canada) was used for all CT data visualization and length measurements.

**Table 1:** Fetal cetacean specimens used in this study.

species	specimen	TL (cm)	% newborn TL length	stage
*Balaenoptera physalus*	USNM 301532	39.0	6.1	2
	USNM 267106	[41]	6.4	2
	USNM 267672	46.8	7.3	3
	USNM 268882	61.5	9.6	4
	USNM 268884	65.0	10.2	4
	USNM 267636	71.5	11.2	4
	USNM 268883	79.1	12.4	5
	USNM 260585	132	20.6	6
	USNM 269156*	[155]	24.2	7
	USNM 269153*	[173]	27.0	7
	USNM 269152*	[208]	32.5	7
	USNM 269151*	[259]	40.5	7
	USNM 268725*	(~290)	45.3	7
	USNM 269154*	[318]	49.7	7
	USNM 269155*	[351]	54.8	7
	USNM 571562*	[500]	78.1	7
*B*. *bonaerensis*	USNM 504716	31.8	13.3	2
	USNM 504718	37.0	15.4	3
	USNM 504715	50.3	21.0	5
*B*. *musculus*	USNM 260581	39.0	5.6	2
	USNM 268885	66.3	9.5	4
	USNM 268001*	(~340)	48.6	7
*Megaptera novaeangliae*	USNM 270342	16.1	4.0	1
	USNM 270341	16.3	4.1	1
	USNM 504979	35.0	8.8	1–2
	USNM 260583	50.4	12.6	4
	USNM 267635	56.4	14.1	4
	USNM 267637	59.2	14.8	5
	USNM 260584	68.8	17.2	5
*Eschrichtius robustus*	USNM 593416*	(~320)	71.1	7
*Eubalaena glacialis*	USNM 500860*	[407]	90.4	7
*Balaena mysticetus*	USNM 571928	130.0	36.1	6
*Stenella attenuata*	USNM 504369	10.3	12.9	1
	USNM 504371	13.1	16.4	1
	USNM 504373	14.6	18.3	1–2
	USNM 504022	19.4	24.3	3
	USNM 504041	20.3	25.4	3
	USNM 504052	27.1	33.9	4
	USNM 504020	30.2	37.8	5
	USNM 504008	35.9	44.9	6
	USNM 504048	75.0	93.8	7
*Globicephala melas*	USNM 241129	13.2	9.6	3
	USNM 012754	22.4	16.2	2
*Phocoena phocoena*	USNM 011227	19.1	27.3	4
	USNM 504982	25.3	36.1	5
	USNM 010764	30.2	43.1	6
	USNM 243600	33.8	48.3	6
	USNM 270633	71.8	102.6	7
*Monodon monoceros*	USNM 241127	32.1	21.4	4
*Pontoporia blainvillei*	USNM 593917	15.2	21.7	2
	USNM 501078	28.7	41.0	6
*Mesoplodon densirostris*	USNM 593918	17.9	9.4	1
*Kogia breviceps*	USNM 504983	26.8	22.3	4–5
	USNM 504985	29.8	24.8	6
	USNM 593919	40.5	33.8	6
*Physeter macrocephalus*	USNM 266891	30.3	8.7	2–3

Total lengths were measured for this study except when the intact specimen was no longer available. Data taken from the collection records are indicated in brackets and rough estimates based on skull size are in parentheses. See S2 Text for detailed descriptions of the developmental stages of the ear. Abbreviations: USNM, National Museum of Natural History (Department of Vertebrate Zoology), Smithsonian Institution, Washington, D.C.; TL = total length.

*Prepared, osteological specimen.

The total length (TL) of all intact specimens were measured in ORS along the dorsal curve from the rostrum to the fluke notch (see [35] for a discussion); cetacean fetuses are often curled, making consistent straight length measurements impossible particularly after fixation and often causing inconsistencies in TL measurements. The relative length of the specimen was obtained by dividing the TL by the reported minimum newborn length of each species [36].

For each CT scanned specimen, the skull was segmented into two separate regions: the tympanoperiotic complex and the rest of the skull, excluding the mandibles. This step used a combination of manual and automatic thresholding techniques, which is more reliable than just automated thresholding techniques [37]. The developmental sequence of the cetacean ears was then divided into 7 stages based on consistent morphological landmarks in the tympanoperiotic complex to facilitate comparisons between taxa (refer to Table 1 and S2 Text).

We did not attempt to obtain physical density values using quantitative computerized tomography techniques [38] because they are likely to be inaccurate and inconsistent between specimens with preservation artifacts. Some of the specimens were collected during whaling operations ~100 years ago and were stored in unknown media before transfer to ethanol, while other specimens were collected within the past ~30 years. The rarity of cetacean fetal specimens makes it extremely difficult to control for such factors. Instead, we measured the relative ossification of the tympanoperiotic complex by calculating a ratio of the maximum CT number (measured in Hounsfield Units) of the tympanoperiotic complex to the maximum CT number of the rest of the skull, excluding the mandibles. The maximum CT number is the most objective, consistent, and repeatable metric for this purpose because the precise boundary between hard versus soft tissues is not as distinct in early, less ossified fetuses, presumably because there is a zone of lower density mesenchyme surrounding each ossification [32].

## Results

**Fig 2:**
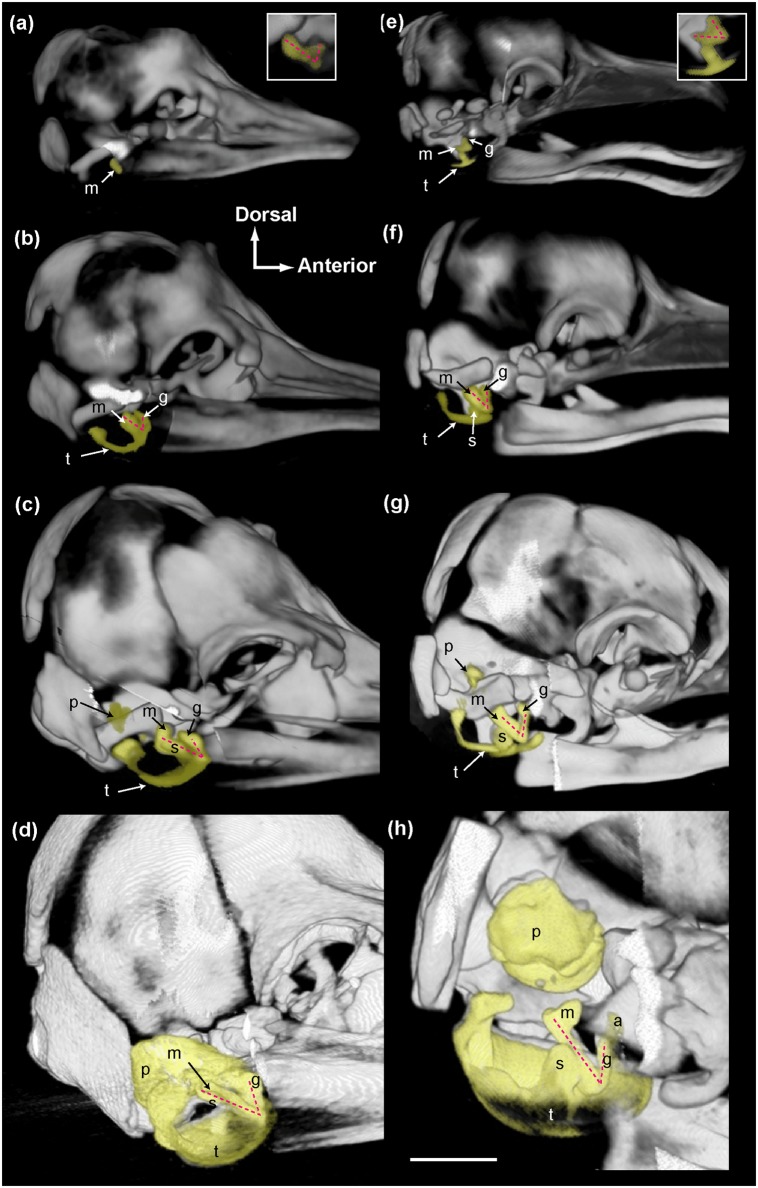
Three-dimensional CT reconstructions illustrating the ontogenetic development of the ears in cetaceans. The tympanoperiotic complex is highlighted in yellow and the right lateral view is shown. Some parts of the skull were digitally removed to expose the ears. The scale bar represents 1 cm for *S*. *attenuata* (a-d) and 2 cm for *B*. *physalus* (e-h) except for the enlarged inserts in (a) and (e), which are twice the size of the corresponding image. The insert in (a) is a flipped image of the left side, in which the goniale is more distinct compared to the right side. Specimens featured in each panel are as follows: a) USNM 504373; b) USNM 504022; c) USNM 504052; d) USNM 504008; e) USNM 301532; f) USNM 267672; g) USNM 268884; h) USNM 260585. Abbreviations: a, accessory ossicle; g, goniale; m, malleus; p, periotic; s, sigmoid process; t, tympanic. The V-shaped malleus-goniale complex is illustrated by the pink dashed line.

The overall pattern of ear development was similar across all taxa in the early stages of development, with readily recognizable mammalian components. Integrating data from various developmental stages across all cetacean species sampled in this study reveals that the acoustic funnel arises from the malleus and the goniale, which form a V-shaped structure facing laterally and slightly anteriorly (Fig. 2). The posterior segment of this V-shape is primarily formed by the prominent malleus, and the goniale represents the anterior segment (refer to S1 Text and Table 1). The goniale is most robust in odontocetes and *Balaenoptera physalus*, and appears as a more slender feature in *B*. *bonarensis*, *B*. *musculus*, and *Megaptera novaeangliae*. This malleus-goniale complex is the only structure present in the lateral wall of the tympanoperiotic complex in early fetal stages and remains the overall most conspicuous, distinct feature of the ears in all early fetal cetaceans (Fig. 2).

In later fetal stages of all odontocetes, the malleus-goniale complex faces anteriorly and slightly dorsally, with the head of the malleus extending dorsally to approach the goniale. In the same ontogenetic range, the goniale extends posteriorly towards the malleus to begin forming the ventral portion of the cone-shaped acoustic funnel. By contrast, the goniale and the malleus remain in a distinct V-formation for our sampled mysticetes and together face laterally. A related character is the orientation of the major axes of the tympanic bullae viewed from the ventral side. The main axes of the right and left tympanic bullae converge anteriorly in all taxa at early stages; however, this pattern changes in all ontogenetically older balaenopterid specimens (evident by TL = 132 cm in *B*. *physalus*; S1 Fig.), where the main axes of the tympanic bullae start to become parallel to the long axis of the skull, which is readily observed in the postnatal condition. In odontocetes and balaenids in our dataset, the main axes of the tympanic bullae converge anteriorly in more mature specimens.

The malleus, goniale, and the tympanic annulus are the earliest structures to form in the tympanoperiotic complex of all cetaceans. The tympanic annulus is not as well-ossified as the malleus and goniale in the early, Stage 2 fetuses. The tympanic annulus originates as a U-shaped structure viewed from the ventral perspective, with the open end of the U facing posteriorly. The medial branch of the U-shaped tympanic annulus elongates posteriorly and laterally and gives rise to the posterior process of the tympanic. The lateral branch of the U-shape fuses to the posterior and ventral margin of the malleus, giving rise to the sigmoid process, which is consistent with previous descriptions [21]. In odontocetes, the sigmoid process grows laterally to form the posterior margin of the cone-shaped acoustic funnel. The sigmoid process of mysticetes also extends laterally, but does not extend to the periotic dorsally; a possible exception is the large *Eubalaena glacialis* specimen (USNM 500860), in which the sigmoid process almost reaches the periotic. Overall, the acoustic funnel is not as cone-shaped in balaenopteroid mysticetes compared to odontocetes.

In the earliest fetal specimens, the tympanoperiotic complex is not well-ossified relative to the rest of the skull. However, the ears rapidly ossify during the early fetal period and become the densest element of the skull (based on CT number) by approximately 20% of the newborn length in mysticetes and approximately 40% of newborn length in odontocetes (S2 Fig.). Mysticetes also reach the latest developmental stage of the ears (Stage 7) earlier than odontocetes relative to newborn length (S3 Fig.). Detailed descriptions of each developmental stage are given in S2 Text.

## Discussion

### Orientation of the acoustic funnel and directionality of sound reception

Our study is the first to identify and depict *in situ* the incipient cetacean acoustic funnel, which is thought to be a critical component of hearing [2, 11, 12]. Although the sound transmission mechanisms of the cetacean middle ear are still debated, the orientation of this acoustic funnel is likely to be functionally important. In odontocetes, the malleus-goniale complex faces more anteriorly and dorsally in later fetal stages (Stage 4 and later; refer to Table 1 and S2 Text), forming part of the ventral floor of the cone-shaped acoustic funnel. The anteriorly oriented malleus-goniale complex, together with the angled tympanics and well-developed sigmoid process, forces the cone-shaped acoustic funnel to face directly into the mandibular foramen and the intramandibular acoustic fats (Fig. 2). The forward-facing orientation of the acoustic funnel is also consistent with the forwardly oriented, very directional receiving beam pattern for high-frequency hearing in odontocetes [39].

In contrast to odontocetes, the malleus-goniale complex in balaenopterid mysticetes faces laterally in later stages. Furthermore, whereas the main axes of the tympanic bullae converge anteriorly in earlier fetuses, just like odontocetes of the same stage, these main axes start to become parallel to the skull in later stages (Stage 6 and later; see S1 Fig.). The lateral orientation of the acoustic funnel in balaenopterids is partly a consequence of this rotation of the tympanic bullae. Although sound reception mechanisms in mysticetes are still unknown, this lateral orientation of the acoustic funnel is consistent with the lateral location of the fat bodies in balaenopterid mysticetes and the recently hypothesized lateral sound reception pathway [13]. However, in mature balaenid mysticetes, the tympanic bullae converge anteriorly, which may indicate a more forward-oriented receiving beam pattern compared to balaenopterid mysticetes. Interestingly, stem cetaceans (or “archaeocetes”), and extinct crown members such as *Aetiocetus weltoni*, a small, toothed fossil mysticete from the late Oligocene, also show anteriorly converging tympanic bullae [40]. Other fossil mysticetes such as *Janjucetus hunderi*, *Chonecetus geodertorum*, *Cetotherium rathkei* and *Herpetocetus transatlanticus* have tympanic bullae that converge anteriorly [41], suggesting that a lateral sound reception pathway may be a relatively new feature both in ontogeny and evolutionary history for mysticetes.

Odontocetes typically vocalize and hear at high frequencies, although previously recorded vocalizations and hearing ranges span a wide range from below 1 kHz to 200 kHz [42, 43]. Mysticetes typically vocalize at low frequencies, ranging from 10 Hz to 28 kHz [42]; hearing ranges are unknown. Some stem cetaceans may have had intermediate hearing ranges, although new evidence suggests that the inner ears of *Zygorhiza* were more sensitive to lower frequency sounds [44]. Given that the wavelengths of a 10 Hz, 1 kHz, 28 kHz, and 200 kHz sound are approximately 150 m, 1.5 m, 5 cm, and 8 mm, respectively, the orientation of the acoustic funnel and the corresponding locations of the fat bodies are likely most relevant for higher frequency sound reception in both odontocetes and mysticetes, as well as stem cetaceans. One hypothesis is that the forwardly oriented acoustic funnel and anterior sound reception pathways in stem cetaceans was a preadaptation for the highly directional, high-frequency hearing of odontocetes. As balaenopterid mysticetes modified their engulfment apparatus for lunge-feeding, their sound reception pathways may have been displaced laterally, unconstrained by the requirements of echolocation.

### Heterochronic processes in ear development for cetaceans

Heterochrony, or change in the onset or timing of organismal development, provides a mechanism for evolution that links ontogeny with phylogeny [45, 46]. Recent studies have shown that heterochronic processes have played a key role in cetacean evolution [47, 48], and our data provide insights into how cetacean ears develop and diverge morphologically in the context of heterochrony. First, the maturation of the ears, both in terms of ossification and morphology, occurs earlier in mysticetes than odontocetes, relative to the newborn body length (S2 and S3 Figs.). This difference can be attributed to the accelerated growth in body size during the middle and later gestational period of mysticetes compared to odontocetes, which results in exceptionally high fetal growth rates to achieve larger body sizes despite relatively short gestational periods [25, 32, 49–53]. Hypothetically, if the earlier, linear growth rate was maintained in mysticetes, the estimated newborn length would be approximately half of the actual newborn length according to whaling industry catch data [50]. If mysticete relative fetal lengths were correspondingly doubled in our dataset, the separation between odontocetes and mysticetes in the rate of ear maturation would disappear.

**Fig 3:**
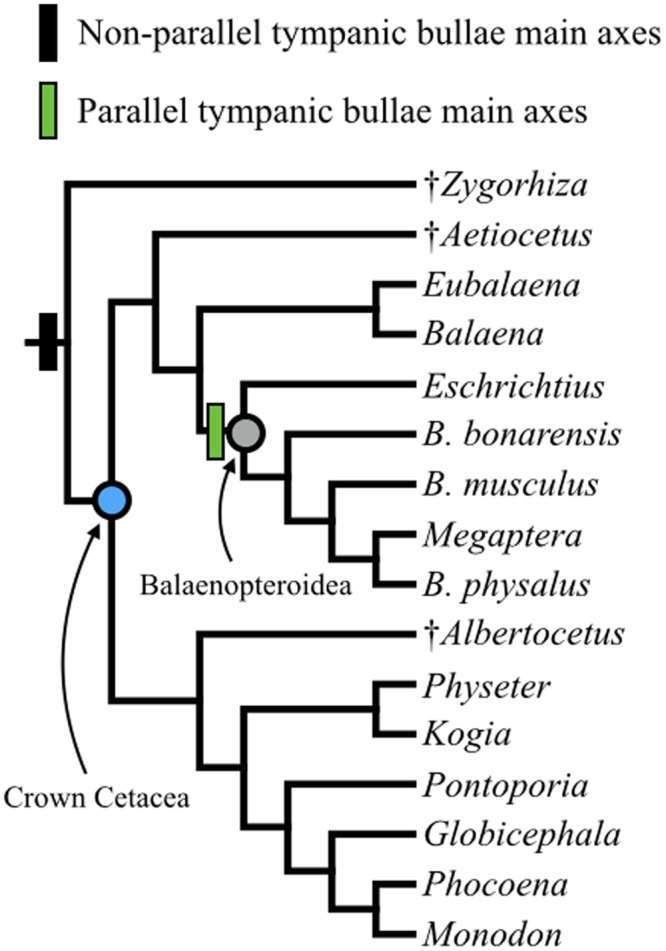
Phylogeny of extant and fossil cetaceans examined in this study. *Zygorhiza*, select extinct crown cetaceans, and odontocetes have tympanic bullae whose main axes converge anteriorly. In contrast, the clade Balaenopteroidea possess tympanic bullae whose main axes are parallel to each other and the skull, correlating with a recently described lateral sound reception pathway, evident at least in balaenopterids. Phylogeny based on [54].

The orientation of the main axes of the tympanic bullae offers another example of heterochrony in cetacean ear development. The condition of parallel-oriented tympanic bullae is a peramorphic trait in Balaenopteridae because the ancestral condition of anteriorly converging tympanic bullae is seen in the earlier ontogenetic stages for our fetal specimens (S1 Fig.; S2 Text; Fig. 3). On the other hand, the relatively large tympanic aperture in mysticetes is a paedomorphic trait. The tympanic aperture of all fetal specimens is relatively large at Stage 6, with its diameter ranging from approximately 24–30% of the tympanic length (all specimens noted as “Stage 6” in Table 1). While this condition is retained in mysticetes, the relative size of the tympanic aperture decreases in Stage 7 odontocete fetuses to 8% of the tympanic length in the *Stenella* specimen and 15% of the tympanic length in *Phocoena* (see Table 1 and S2 Text). Thus, our expansive ontogenetic series of fetal specimens allows us to understand how the diverse adult morphologies arise in different cetacean lineages.

### Evolutionary implications

The lack of non-keratinized soft cranial tissue preservation in the fossil record prevents clear identification of the acoustic funnel in extinct cetaceans, but the phylogenetic distribution of tymapanoperiotic traits observed in our dataset allows for several inferences. First, the early development of the acoustic funnel from the goniale and malleus in both mysticetes and odontocetes points to the acoustic funnel being shared in all crown Cetacea; thus, its antiquity is minimally placed near the Eocene-Oligocene boundary (~34–33 million years ago), the likeliest timeframe for the origin of this clade [54] (Fig. 3). It is unclear whether stem cetaceans (or “archaeocetes”) shared this trait, especially because of the tremendous morphological transformations in the ear during this part of cetacean history [17–20]. Based on the orientation of the tympanic bulla in articulated tympanoperiotics of basilosaurids and protocetid-grade stem cetaceans (*Basilosaurus isis* and *Gaviacetus razai*, with the possible exception of *Indocetus ramani* [18]), a forwardly oriented acoustic funnel may have been present in many crown-ward stem cetaceans. Remingtoncetids (*Andrewsiphius* and *Remingtonocetus*) may have possessed an acoustic funnel; the large mandibular foramen in this lineage would have provided the anatomical space to house intramandibular acoustic fats, providing a lipid conduit to the ears [20]. Well-preserved fossils show patent bony tissue configurations in stem cetaceans similar to those seen in crown cetaceans, including anteriorly converging tympanic bullae, in *Remingtonocetus harudiensis* [55]. Pakicetids, the most basal lineage of cetaceans, lacked specializations for aquatic hearing [17, 19, 20], and presumably therefore lacked an acoustic funnel. Ultimately, resolving the transition between sound reception via the ear canal and alternative sound reception pathways that evolved in crown cetaceans, and some stem cetaceans, will require more paleontological data: especially from ontogenetic stages that are usually poorly preserved [20, 56].

Our results have important implications for the evolution of hearing and feeding within crown Cetacea, as discussed above. Ancestrally, crown cetaceans appear to have tympanic bullae whose main axes are converging anteriorly [41], which orients the acoustic funnel anteriorly or anterolaterally. In contrast, the main axes of the tympanic bullae are oriented parallel to each other and to the skull in gray whales and rorquals, which together form a clade (crown Balaenopteroidea, see [41, 57, 58]). This distribution implies that laterally oriented acoustic funnels accommodate the large excursions of the mandible in rorquals [59, 60], where the extreme rotation of the temporomandibular joint (TMJ) may preclude an anteriorly directed acoustic pathway. Details of TMJ and the mandibular symphyses in gray whales remain unclear (see [54], contra [61]), although both features appear broadly similar to those reported in rorquals [60]. The proposed phylogenetic nesting of gray whales within Balaenopteridae, based on weak molecular support [41, 58], implies that gray whales lost major specializations for lunge-feeding, although it does not bear on the innovation of lateral sound reception at the node of Balaenopteroidea.

### Caveats

This study is unique in its broad taxonomic coverage and large number of specimens because CT techniques provide non-invasive ways to examine rare, irreplaceable museum specimens. However, CT has a limited resolution and we were unable to detect early features of the tympanoperiotic complex in specimens with TL < 16 cm. Furthermore, the morphology of structures that are not well-ossified is difficult to see on the CT scans, which rely on differential X-ray attenuation through tissues to create contrast. MicroCT and magnetic resonance microscopy techniques have been applied to at least three very early cetacean fetuses [31, 32], and will provide additional non-destructive insights into cetacean development in the future. However, these techniques are still limited in the ability to distinguish between early cartilage and connective tissue. Thus, a combination of conventional techniques (i.e., histology or classic dissection) and non-invasive modern techniques are necessary for complete anatomical analyses [33].

We could not control for any potential preservation artifacts of the fluid-preserved specimens, which may have been fixed in unknown solutions before transfer to ethanol. However, our results are consistent with previous morphological descriptions of equivalent stages and taxa, including data from dissections, cleared and stained specimens, histology, and other non-invasive techniques (see S1 Text and S2 Text).

Lastly, tremendous progress has been made in the study of cetacean sound reception over the past several decades [43]. Nonetheless, there are still large uncertainties regarding the exact mechanisms of the cetacean middle ear, the role of the acoustic funnel, and sound reception in mysticetes. While these issues present challenges and limitations for interpretation of new data, they also underscore the importance of using a variety of approaches and methods, such as our ontogenetic study leveraging archived museum specimens.

## Conclusions

By following ontogenetic series of cetacean fetuses across a broad range of taxa, we found that the unique acoustic funnel into the cetacean ear arises from a V-shaped feature formed by the malleus and goniale early in fetal development. The malleus-goniale complex is a well-ossified, persistent, and conspicuous feature of the fetal cetacean ear. In mature odontocetes, the malleus-goniale complex forms the ventral portion of the cone-shaped acoustic funnel [11, 12], to which the intramandibular acoustic fats attach anteriorly [2,12]. In mysticetes, a large body of fat hypothesized to be a lateral sound reception pathway in at least some balaenopterid mysticetes [13] inserts into the tympanoperiotic complex inside the opening created by the V-shaped malleus-goniale complex. Phylogenetic mapping of these traits and visual inspection of well-preserved fossils show that acoustic funnels likely provided a pathway for sound reception in Oligocene crown cetaceans (e.g., *Aetiocetus cotylalveus* USNM 25210 and *Albertocetus meffordorum* USNM 525001 from NMNH Paleobiology collections), and may potentially be inferred in some Eocene stem cetaceans as well.

We found that the orientation of the acoustic funnel correlates with previously described sound reception pathways for each clade, although cetacean middle ear mechanisms are still debated and key experimental evidence is lacking in mysticetes due to the logistical as well as legal challenges of working with these large marine mammals. The acoustic funnel faces anteriorly in all fetal cetaceans and remain this way in mature odontocetes and balaenids, while the acoustic funnel rotates laterally later in ontogeny in balaenopterid (and likely, all balaenopteroid) mysticetes. Fossil cetaceans possess anteriorly oriented acoustic funnels, suggesting that crown cetaceans mainly employ ancestral sound reception pathways inherited from stem cetaceans, except for rorquals and gray whales. The examination of fetal specimens provides insight into the developmental basis for major evolutionary innovations, including the aquatic sound reception pathways of cetaceans.

## Supporting Information

S1 Text The goniale and accessory ossicle. (DOCX)

S2 Text Developmental stages of the tympanoperiotic complex. (DOCX)

S1 Fig Three-dimensional reconstructions of the tympanoperiotic complex in ventral view. (a-c) *S*. *attenuata*; (d) *B*. *borealis*; (e) *B*. *musculus*; (f) *M*. *novaeangliae*; (g-i) *B*. *physalus*. The tympanoperiotic complex is highlighted in yellow and the main axes of the tympanic bullae are approximated by the dashed pink line. Specimens featured in each panel are as follows: a) USNM 504052; b) USNM 504008; c) USNM 504048; d) USNM 504718; e) USNM 268885; f) USNM 267637 (yellow highlight for tympanic only); g) USNM 268884; h) USNM 268883; i) USNM 260585.
(TIF)

S2 Fig The ossification of the tympanoperiotic complex through ontogeny. All CT scanned specimens are represented in this figure. The relative density of the ears is obtained by dividing the maximum CT number of the tympanoperiotic complex by the maximum CT number of the rest of the skull. TL = total length. The newborn length of each species was obtained from [36].
(TIF)

S3 Fig Developmental stages of the ears as functions of relative length (left panel) and total length (right panel) for all specimens. Refer to S2 Text for descriptions of each stage.
(TIF)

S4 Fig Osteological specimens representing Stage 7 mysticete ears. (a) *B*. *physalus*, USNM 269156. (b) *Eschrichtius robustus*, USNM 593416. Scale bar = 2 cm.
(TIF)
