# Supplementary material for: Early Development and Orientation of the Acoustic Funnel Provides Insight into the Evolution of Sound Reception Pathways in Cetaceans
Source: PLoS One. 2015 Mar 11;10(3):e0118582. doi: 10.1371/journal.pone.0118582 (PMC4356564; doi:10.1371/journal.pone.0118582)
Supplement: S1 Text — (DOCX) [file pone.0118582.s001.docx]

Supporting Information for

**Early development and orientation of the acoustic funnel provides insight into the evolution of sound reception pathways in cetaceans**

by Maya Yamato and Nicholas D. Pyenson

**Text S1. The goniale and accessory ossicle**

Some confusion exists regarding the goniale in cetaceans. According to Fleischer [9], the goniale is a bony element fused to both the malleus and tympanic bone in the primitive mammalian condition. In some mammals, the goniale has been reduced to the anterior process of the malleus. This situation is seen in humans, in which the goniale becomes prominent at the end of the embryonic period and is an independent ossification which eventually gives rise to the anterior process of the malleus [62]. The cetacean goniale, which is fused to both the malleus and tympanic bone in adults, is closer to the primitive mammalian condition. In other mammals, the fusion of the goniale with the tympanic ring is a feature correlated with high frequency hearing, along with a small middle ear volume, small tympanic membrane, and strong middle ear muscles [63, 64]. Fleischer states that the goniale is longer in mysticetes than in odontocetes, but the malleus-incus complex is otherwise similar to the odontocete condition [9]. An excellent discussion on the evolution of the middle ear in terrestrial mammals, including illustrations of the malleus and goniale, is found in [65], and [66] provides a thorough re-examination of the middle ear in Carnivora.

Another related enigmatic structure is the cetacean accessory ossicle. The accessory ossicle is a structure that is prominent in all extant odontocetes, fusing to the tympanic bulla anterior to the sigmoid process and forming the anterior connection between the tympanic and periotic bones [14]. The odontocete accessory ossicle is homologous to the embryonic accessory ossicle of ungulates, which is incorporated into the bullar body as the process tubarius in adult ungulates instead of being maintained as an independent ossification [17].

While extant mature mysticetes do not have an accessory ossicle, it can be found in fetal mysticetes [21]. The odontocete accessory ossicle may be a neotenic feature, based on the resemblance between the fetal mysticete accessory ossicle and the mature odontocete accessory ossicle [17]. The mysticete accessory ossicle forms the anterior pedicle of the tympanic later in development [14, 21].

This study follows the terminology of Ridewood [21], who stated that the accessory ossicle does not exist in any of his specimens (*Megaptera novaeangliae* with straight lengths = 15.2, 40.6, and 68.6 cm, and a 119.4 cm-long *Balaenoptera borealis*) except in the most mature specimen, a 193-cm long *Balaenoptera musculus* with a recognizable, almost mature tympanic bulla morphology. In this blue whale specimen, he describes the accessory ossicle as a “separate flake of bone” which later becomes the anterior pedicle of the tympanic. In our study, the accessory ossicle could not be identified in earlier fetal mysticetes, but is seen as an independent ossification just anterior to the goniale in the 132 cm *Balaenoptera physalus* specimen (USNM 260585, Fig. 2h).

There is no mention of the accessory ossicle in Eales’s description [23] of a 13.7 cm-long *Monodon monoceros*, although he identified and described the goniale as a small membrane bone between the cartilaginous malleus and the antero-lateral branch of the “U” shaped tympanic crescent. Confusingly, Kesteven [22], in his description of a *Delphinus delphinus* skull measuring 7.8 cm, stated that there are 2 accessory ossicles, which are both flat flakes of bone attached to the antero-lateral margin of the body of the malleus, above the “inferior process” (likely referring to the anterior process). He equated Ridewood [21]’s accessory ossicle to his anterior accessory ossicle and Ridewood [21]’s goniale to his posterior accessory ossicle.

The accessory ossicle is also addressed by Moran et al. [28], which stated that the accessory ossicle is one of the earliest features to ossify in the *Stenella* ear. Based on Moran et al. [28]’s description of the accessory ossicle as a densely ossified structure that overlies, and is fused to, Meckel’s cartilage, which is continuous with the cartilaginous malleus in the 15.5 cm *Stenella* fetus, we surmise that Ridewood [21]’s “goniale”, Kesteven [22]’s “posterior accessory ossicle”, and Moran et al. [28]’s “accessory ossicle” refer to the same feature in early cetacean fetuses, which we refer to as the “goniale” in this study. This situation leaves open the ontogeny of the accessory ossicle as defined by Mead and Fordyce [14] in odontocetes. What we interpret as an incipient accessory ossicle could be seen in two of our odontocete specimens: *Pontoporia blainvillei* (USNM 593917) and *Kogia breviceps* (USNM 504983), which shows a small, independent ossification located between the malleus and goniale, which is a different location compared to the *B. physalus* in Figure 2h.

**Additional references**

1. Vasquez JF, Velasco JR, Collado J. A study of the os goniale in man. Cells Tissues Organs. 1991;142(2): 188-192.
2. Hunt RM, Korth WW. The auditory region of dermoptera: Morphology and function relative to other living mammals. Journal of Morphology. 1980;164(2): 167-211.
3. Lange S, Stalleicken J, Burda H. Functional morphology of the ear in fossorial rodents, *Microtus arvalis* and *Arvicola terrestris*. Journal of Morphology. 2004;262(3): 770-779.
4. Luo Z. Developmental patterns in Mesozoic evolution of mammal ears. Annual Review of Ecology, Evolution, and Systematics. 2011;42: 355-380.
5. Wible JR, Spaulding M. A Reexamination of the Carnivora Malleus (Mammalia, Placentalia). PloS one. 2012;7(11): e50485.
