# Supplementary material for: Early Development and Orientation of the Acoustic Funnel Provides Insight into the Evolution of Sound Reception Pathways in Cetaceans
Source: PLoS One. 2015 Mar 11;10(3):e0118582. doi: 10.1371/journal.pone.0118582 (PMC4356564; doi:10.1371/journal.pone.0118582)
Supplement: S2 Text — (DOCX) [file pone.0118582.s002.docx]

Supporting Information for

**Early development and orientation of the acoustic funnel provides insight into the evolution of sound reception pathways in cetaceans**

by Maya Yamato and Nicholas D. Pyenson

**Text S2. Developmental stages of the tympanoperiotic complex**

***Stage 1*. (Specimens available for *Megaptera, Stenella, Mesoplodon*)**

The tympanoperiotic complex is indistinct in the CT scans of the earliest fetuses examined (16-cm long *Megaptera* specimens and 10-13 cm-long *Stenella*; specimen numbers are given in Table 1). The malleus on both sides and part of the left goniale is discernible in the 14.6 cm-long *Stenella* specimen (Fig. 2a), which is in between Stage 1 and Stage 2. Previous work has shown that a cartilaginous periotic element with a distinct, fluid-filled cochlea is already present in a *Stenella* specimen with a crown-rump length of 3.8 cm, but the boundaries between cartilage and the surrounding soft tissue could not be resolved even with magnetic resonance microscopy [31]. Another study of cleared and stained early *Stenella* fetuses showed a horseshoe-shaped tympanic annulus for a specimen with a TL of 8.5 cm, but it is a very thin, barely ossified element [28] that is not visible in the CT images. No component of the tympanoperiotic complex is visible in the microCT data presented for a 13 cm-long *Balaenoptera musculus* fetus [32].

***Stage 2*. (Specimens available for *Balaenoptera physalus, B. bonarensis, Megaptera, Stenella, Physeter, Pontoporia*)**

The malleus, goniale, and the tympanic annulus are the only structures that are detected in the tympanoperiotic complex at Stage 2. The malleus and goniale form a “V” shape facing laterally and slightly anteriorly (Fig. 2). The posterior segment of the “V” is primarily formed by the malleus, and the goniale represents the anterior segment. This “V” formation is the only structure present in the lateral wall of the tympanoperiotic complex in early fetal stages and persists as a well-ossified, distinct feature of the ears in all fetal cetaceans except for the most mature odontocete fetuses. Exceptions are the *B. bonarensis* and *Megaptera* specimens at this stage, in which the goniale is only faintly visible and the “V” shape is not as distinct. However, the tympanic annulus is visible in these specimens.

At least in the Balaenopteridae and Eschrichtiidae, the tympanic annulus originates as a “U” shaped structure viewed from the ventral perspective, with the open end of the “U” facing posteriorly. The anterior, outer margin of the “U” is somewhat pointed. The 30.3 cm-long *Physeter* specimen indicates that this pattern also holds for *Physeter*, although this specimen is in a transitional state between Stage 2 and 3.

What we interpret as the accessory ossicle is visible in the *Pontoporia* specimen as a small, rounded, independent ossification dorsal to the malleus and the goniale.

***Stage 3.* (Specimens available for *B. physalus, B. bonarensis*, *Stenella, Physeter, Globicephala*)**

The tympanic annulus is not as clear in the odontocete specimens, but it is also somewhat “U” shaped from the ventral view. The anterior, outer margin of the “U” is extending to form the anterior process of the tympanic in the *Stenella* and *Globicephala* specimen.
 In all Stage 3 specimens, the medial branch of the “U” shaped tympanic annulus elongates posteriorly and laterally (see Fig. S1) and gives rise to the posterior process, which is just starting to form. At least in mysticetes, the lateral branch of the “U” eventually fuses to the posterior and ventral margin of the malleus and will give rise to the sigmoid process. The feature that will develop into the sigmoid process is already visible in the Stage 3 *B. physalus* specimen.

From the ventral perspective, the tympanic bulla has not yet developed into a recognizable, adult-like form except in *B. bonarensis*. However, in *B. bonarensis,* the goniale is still inconspicuous.

***Stage 4*. (Specimens available for *B. physalus, B. musculus, Megaptera, Stenella, Phocoena, Monodon, Kogia*)**

The periotic ossifies later than the tympanic, and is first detected in Stage 4. The periotic and tympanic are not in contact with each other.

The anterior, outer margin of the “U” shaped tympanic annulus extends anteriorly to form the anterior process of the tympanic bone, which is present in all Stage 4 specimens except for *Megaptera*. The medial wall of the tympanic bone has not yet formed and the tympanic bulla has not assumed a recognizable, adult-like form from the ventral perspective except in *B. physalus* (and *B. bonarensis*).

The involucrum is not present in any taxa at this stage. The incus and stapes could not be detected in any mysticete specimens at Stage 4, but the incus can be found between the malleus and the emerging periotic in all Stage 4 odontocetes. In *B. musculus* and *Megaptera*, the goniale is present but is still not yet robust at this stage*.*

In the odontocetes, the “V” formed by the malleus and goniale is starting to face more dorsally than in Stage 3. The head of the malleus is extending dorsally to approach the goniale (Fig. 2c). At the same time, the goniale is extending posteriorly towards the malleus to begin forming the cone-shaped acoustic funnel (named the “sound-funnel” by Boenninghaus [11] and “ear trumpet” by Cranford et al [12]). In mature specimens, this cone-shaped structure is filled by acoustic fats extending from the mandibular foramen [2, 12]. What is presumed to be the accessory ossicle is visible in the *Kogia* specimen as a rounded element located in between the malleus and goniale.

In contrast to the situation in odontocetes, the goniale and malleus remain in a distinct “V” formation in the mysticetes. Instead of rotating dorsally, the “V” is rotating slightly laterally in mysticetes.

The sigmoid process is visible in all Stage 4 specimens, although it is configured differently in the odontocetes and mysticetes. In mysticetes, the lateral margin of the U-shaped tympanic annulus fuses to the ventral and posterior margin of the malleus and continues to grow as a rounded, knob-like process. In odontocetes, the early origin of the sigmoid process is less clear but it appears as a thin, shelf-like projection coming off of the lateral margin of the malleus.

The major axes of the tympanic bullae are converging anteriorly in all Stage 4 specimens.

***Stage 5.* (Specimens available for *B. physalus, M. novaeangliae, Stenella, Kogia*)**

The periotic grows rapidly between Stage 4 and 5. The periotic is just starting to form in the 61.5 cm-long *B. physalus* fetus (Stage 4; Fig. 2g) and is still a very small ossification in the 71.5 cm-long specimen (still Stage 4), but in the 79.1 cm-long (Stage 5) specimen, the periotic is well-developed and is approximately the same size as the tympanic bulla. Similarly, in *Stenella*, the periotic is a miniscule structure in the 27.1 cm-long (Stage 4) specimen but is almost the same size as the tympanic and is well-developed in the 30.2 cm-long (Stage 5) specimen. The periotic and tympanic are not in contact with each other in any of the Stage 5 specimens.

The incus and the stapes ossify later than the malleus and are difficult to visualize in the CT scans due to their small sizes. In *Megaptera*, Ridewood [21] states that they are not yet ossified in a 68.6 cm-long fetus. The footplate of the stapes is clearly visible in our 68.8 cm-long *Megaptera* specimen, but no other component of the stapes or incus could be detected. In *B. physalus*, what appears to be the stapes, and possibly the incus, are distinguishable in the Stage 5 fetus. All ossicles are detectable in the Stage 5 odontocete specimens, although they are less clear in the transitional *Kogia* specimen.

The anterior, medial, and posterior margins of the tympanic bulla have developed enough to give the tympanic a recognizable shape in all taxa, although the tympanic is not as well-formed in the *Megaptera* specimens. The goniale is now also a prominent feature in *Megaptera*, but is still not robust in *B. bonarensis*.

In odontocetes, the “V” shape formed by the malleus and the goniale is starting to close as the malleus and goniale come together. In mysticetes, the head of the malleus is also becoming distinct as it protrudes anteriorly and slightly dorsally towards the goniale. However, the malleus and goniale maintain their separation and still form a distinct “V” shape.

The conical process is not visible in any of the Stage 5 specimens.

The main axes of the tympanic bullae are still converging anteriorly in all specimens, although to a lesser degree in the Balaenopterid mysticetes.

***Stage 6.* (Specimens available for *B. physalus*, *Balaena, Stenella, Phocoena, Kogia*)**

In mysticetes, the tympanic becomes much larger than the periotic in lateral view (the anterior and posterior processes of the periotic have not yet formed). In odontocetes, the periotic and the tympanic are still similar in size. The tympanic and periotic are in contact with each other in the odontocete specimens, although they are not fused (indicated by a very small gap in the axial CT scans). In the mysticete specimens, no part of the tympanic and periotic contact each other.

The tympanic aperture is large in both odontocete and mysticete specimens, with the diameter of the tympanic aperture ranging from approximately 24-30% of the tympanic length.

The conical process is visible in the 132 cm-long *B. physalus* specimen, but not in the 130 cm *Balaena* specimen.

The main axes of the tympanic bullae are no longer converging anteriorly in the *B. physalus* specimen and are instead parallel to the main axis of the skull. The odontocete tympanic bullae, as well as the *Balaena* tympanic bullae, are still converging anteriorly.

***Stage 7.* (Specimens available for *Stenella*, *Phocoena,* *Eschrichtius,* *B. musculus,* *B. physalus*)**

The conical process is visible in all Stage 7 specimens that are available for this study. In odontocetes, the tympanic aperture has separated into 2 components: the genuine tympanic aperture and the ductus petro-tympanicus, or hiatus epitympanicus [2, 14]. Both components are very small relative to the Stage 6 specimens, and the diameter of the genuine (lower) tympanic aperture is 8% of the tympanic length in the 75 cm-long *Stenella* and 15% of the tympanic length in the 72 cm-long *Phocoena* specimen (compared to 29% in both the 36 cm-long *Stenella* and 30 cm-long *Phocoena*, and 26% in the 34 cm-long *Phocoena*). In contrast, the Stage 7 *B. physalus* mysticete specimen has a tympanic aperture diameter that is 32% of the tympanic length (compared to 27% in the previous stage).

In the 155 cm-long *B. physalus* (osteological specimen), the posterior process of the tympanic bone is in contact with the periotic but is not fused to it. Of the incus and stapes, only the footplate of the stapes is present; the incus may have been lost during preparation. The anterior and posterior processes of the periotic bone have not yet formed. In the 259 cm-long *B. physalus* specimen, the goniale has extended dorsally and is in contact with the periotic bone. Although the two elements are not yet fused, this extension of the goniale is forming the anterior pedicle connecting the tympanic and periotic bones. In the *B. physalus* specimens larger than 290 cm, the anterior pedicle is much broader, potentially due to contributions from the accessory ossicle in the anterior portion.

The sigmoid process is enlarged in all specimens and is projecting laterally from the malleus. This is particularly pronounced in odontocetes, where the prominent sigmoid process forms the posterior wall of the cone-shaped acoustic funnel. The projecting sigmoid process also makes the cone face directly anteriorly in odontocetes, pointing into the mandibular foramen. In mysticetes, the equivalent feature is facing laterally instead of anteriorly.

By Stage 7, the tympanoperiotic complex is the densest element of the skull in all taxa examined (relative density of 1.47-1.59). This is consistent with previous studies that have shown that the tympanoperiotic complex is already in a mature form and is the densest element of the skull in perinatal cetaceans [29, 33, 34].
