# Supplementary figures and images for: Early Development and Orientation of the Acoustic Funnel Provides Insight into the Evolution of Sound Reception Pathways in Cetaceans
Source: PLoS One. 2015 Mar 11;10(3):e0118582. doi: 10.1371/journal.pone.0118582 (PMC4356564; doi:10.1371/journal.pone.0118582)

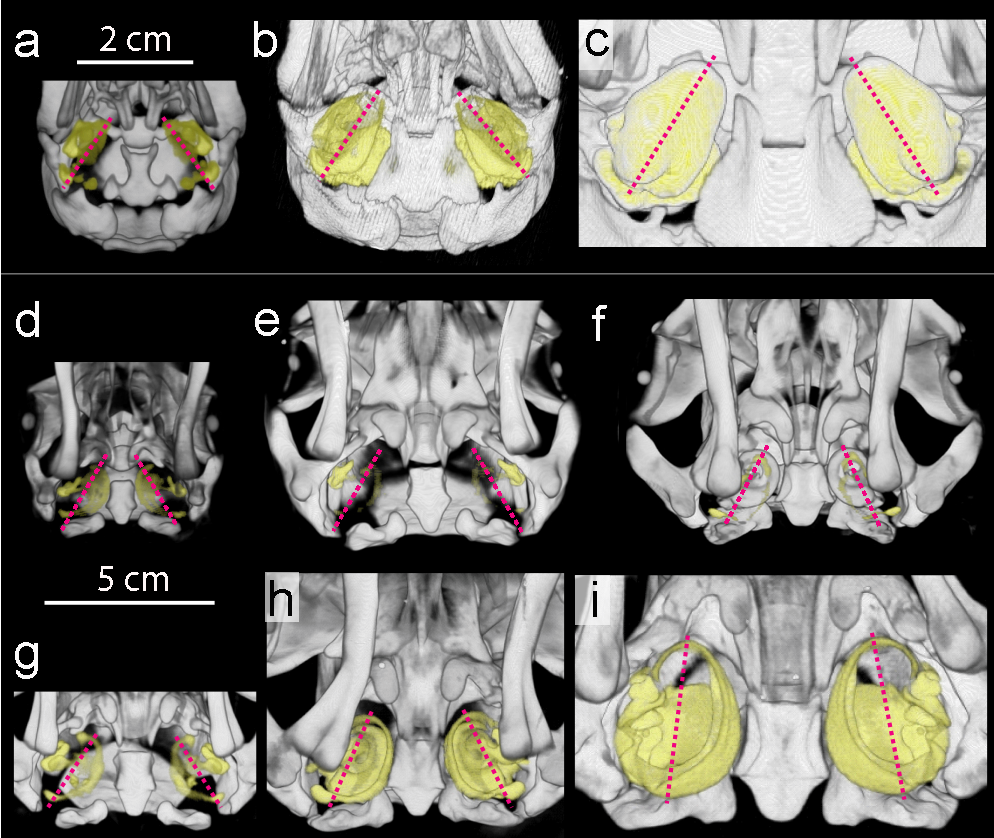

Supplement: S1 Fig — (a-c) S. attenuata; (d) B. borealis; (e) B. musculus; (f) M. novaeangliae; (g-i) B. physalus. The tympanoperiotic complex is highlighted in yellow and the main axes of the tympanic bullae are approximated by the dashed pink line. Specimens featured in each panel are as follows: a) USNM 504052; b) USNM 504008; c) USNM 504048; d) USNM 504718; e) USNM 268885; f) USNM 267637 (yellow highlight for tympanic only); g) USNM 268884; h) USNM 268883; i) USNM 260585. (TIF) [file pone.0118582.s003.tif]

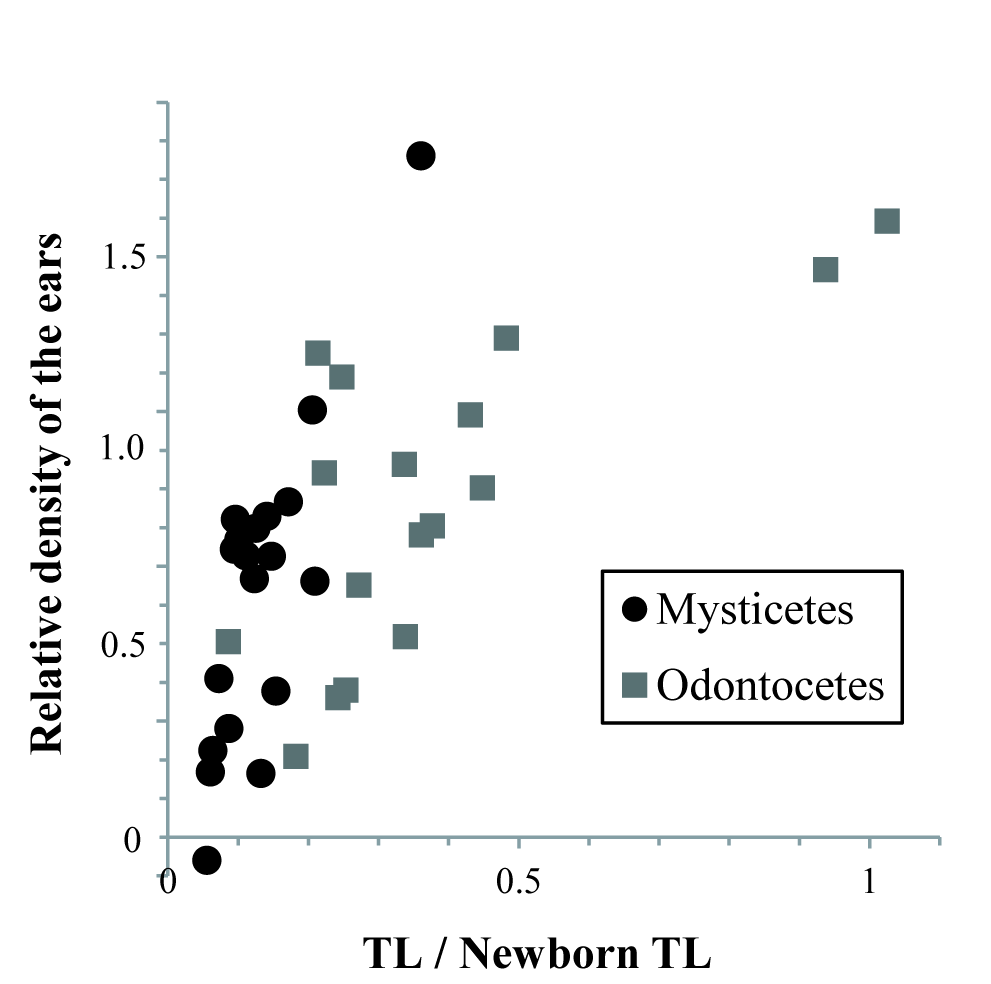

Supplement: S2 Fig — All CT scanned specimens are represented in this figure. The relative density of the ears is obtained by dividing the maximum CT number of the tympanoperiotic complex by the maximum CT number of the rest of the skull. TL = total length. The newborn length of each species was obtained from [36]. (TIF) [file pone.0118582.s004.tif]

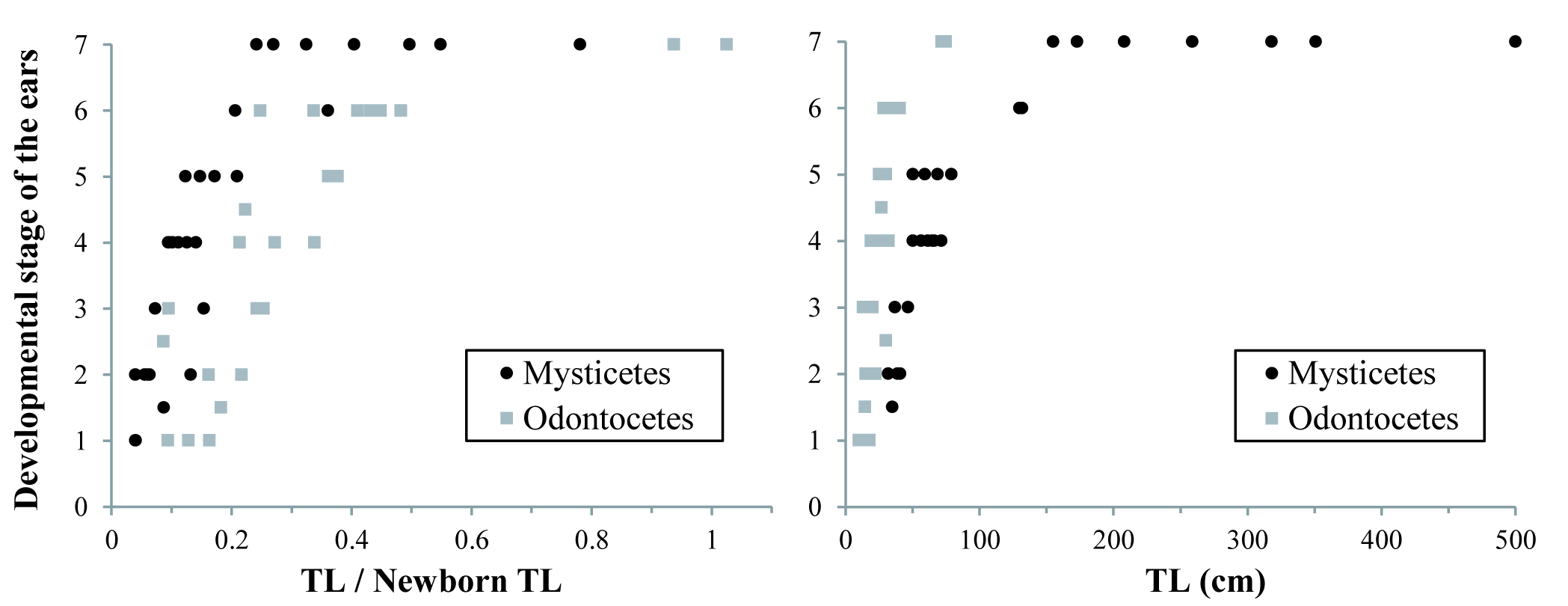

Supplement: S3 Fig — Refer to S2 Text for descriptions of each stage. (TIF) [file pone.0118582.s005.tif]

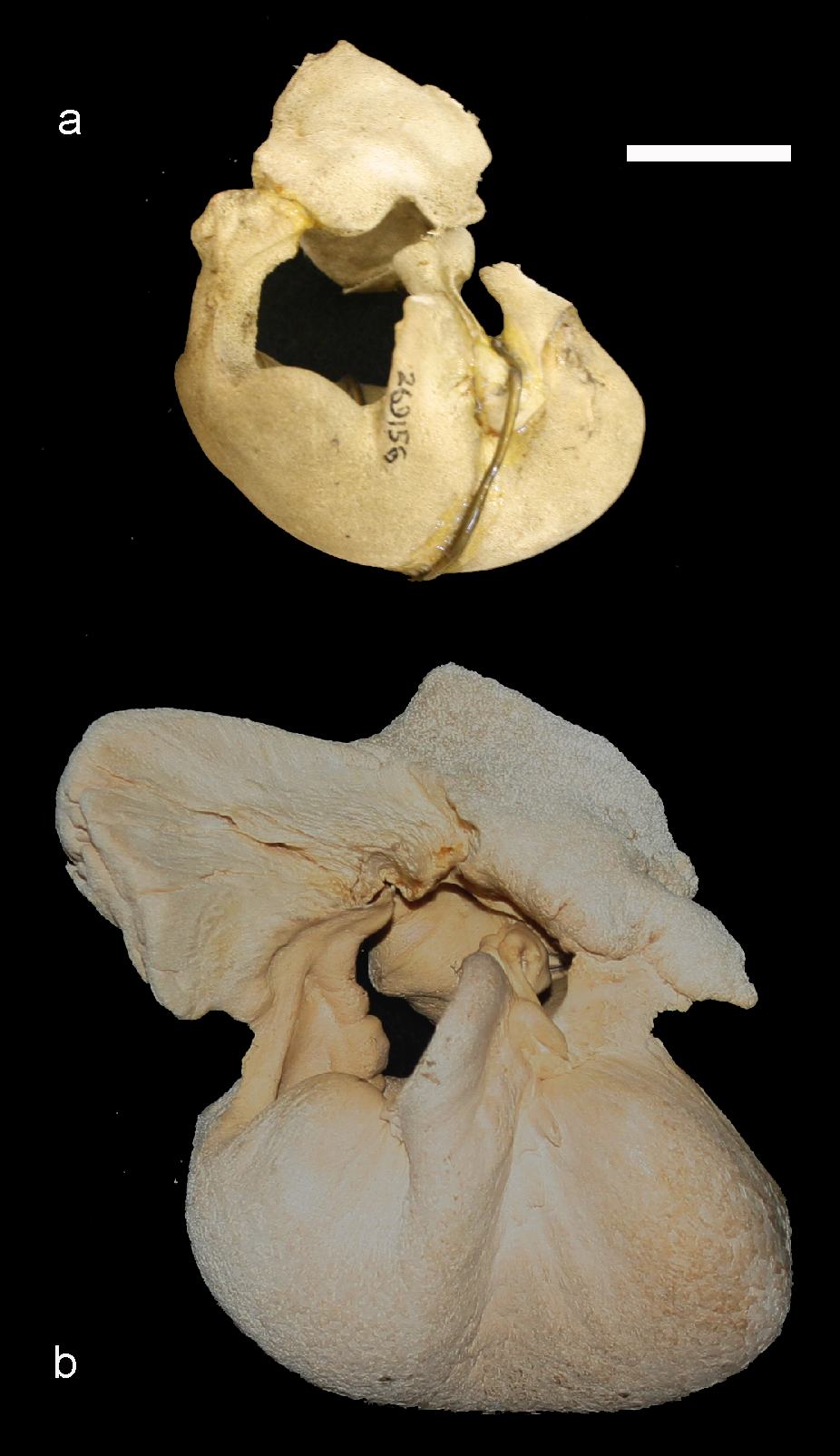

Supplement: S4 Fig — (a) B. physalus, USNM 269156. (b) Eschrichtius robustus, USNM 593416. Scale bar = 2 cm. (TIF) [file pone.0118582.s006.tif]
